# Genetic and environmental factors influencing the contents of essential oil compounds in *Atractylodes lancea*

**DOI:** 10.1371/journal.pone.0217522

**Published:** 2019-05-28

**Authors:** Takahiro Tsusaka, Bunsho Makino, Ryo Ohsawa, Hiroshi Ezura

**Affiliations:** 1 Graduate School of Life and Environmental Sciences, University of Tsukuba, Tsukuba, Ibaraki, Japan; 2 Botanical Raw Materials Production Department 2, Tsumura & Co., Ami, Ibaraki, Japan; 3 Botanical Raw Materials Research Laboratories, Tsumura & Co., Ami, Ibaraki, Japan; 4 Faculty of Life and Environmental Sciences, University of Tsukuba, Tsukuba, Ibaraki, Japan; Institute for Biological Research, SERBIA

## Abstract

Rhizomes of *Atractylodes lancea* are used in traditional Japanese medicine (Kampo) and Chinese medicine to treat numerous diseases and disorders because they contain many pharmacologically active compounds. The major active compounds in *A*. *lancea* are essential oil compounds such as β-eudesmol, hinesol, atractylon, and atractylodin. The contents of the compounds in *A*. *lancea* exhibit high variability depending on their habitat. We cultivated clonal lines of *A*. *lancea* in different years (2016, 2017) and different locations (Hokkaido, Ibaraki) to investigate the influence of genetic and environmental factors on the contents of major compounds, namely, β-eudesmol, hinesol, atractylon, and atractylodin. Broad sense heritability of β-eudesmol, hinesol, atractylon, and atractylodin contents were 0.84, 0.77, 0.86, and 0.87, respectively. The effects of interannual variability on the contents of the compounds were lower than those of genotype. In addition, the cultivated environmental factors were assessed by different locations, and the correlations between Hokkaido and Ibaraki grown plants based on β-eudesmol, hinesol, atractylon, and atractylodin contents were 0.94, 0.94, 1.00, and 0.83, respectively. The results suggest that the contents of β-eudesmol, hinesol, atractylon, and atractylodin in *A*. *lancea* are largely influenced by genetic factors, and clonal propagation could be an effective strategy for obtaining populations with high contents of essential oil compounds. Furthermore, the contents of β-eudesmol, hinesol, atractylon, and atractylodin in *A*. *lancea* exhibited few correlations with rhizome yields. *A*. *lancea* cultivars with not only high contents of essential oil compounds but also high rhizome yield could be developed through selective breeding.

## Introduction

*Atractylodes lancea* De Candolle (*Compositae*) is a medicinal plant that is distributed in East Asia, mainly in central China [1]. Dried rhizomes of *A*. *lancea* are prescribed in Japanese and Chinese herbal medicines as the crude drugs [2, 3]. In Japan, the crude drug is called “So-Jutsu,” whereas in China, it is called “Chang-zhu” [4]. The rhizomes of *A*. *lancea* have mainly been used for digestive disorders and body fluid imbalance [5, 6]. Major active ingredients in *A*. *lancea* were essential oil compounds, namely, sesquiterpenoids (β-eudesmol, hinesol, atractylon) and polyacetylene (atractylodin) [7]. The sesquiterpenoid compounds have been reported to have anti-ulcer activity, while atractylodin has been reported to ameliorate the delayed gastric emptying [8, 9]. Based on pharmacological studies, such compounds are considered important bioactive ingredients in *A*. *lancea* [5]. However, the drugs distributed in the market have hugely varied in contents of the major essential oil compounds, posing a challenge for the cultivation of *A*. *lancea* with constant quality [10]. Therefore, it is critical to investigate the factors influencing the variation in the contents of essential oil compounds in *A*. *lancea* to keep pharmacological activities constant in many traditional prescriptions containing the crude drug [10].

Generally, phenotypic variation is a product of both genetic and environmental variation [11]. The qualities of *A*. *lancea* samples are closely associated with their habitat, and the contents of essential oil compounds in *A*. *lancea* vary considerably based on geographical factors. However, it remains unclear whether the geographical variations are based on genetic or environmental factors [12, 13].

Some studies have reported that the activation of phytohormones such as jasmonic acid and abscisic acid through symbiosis with several endophytes induces the production of the essential oil compounds in *A*. *lancea* [14, 15]. In addition, Yuan *et al*. [16] reported that soil acidity stimulated the accumulation of essential oils in *A*. *lancea*, particularly β-eudesmol, by influencing the concentrations of phytohormones such as abscisic acid. Such results suggest that the contents of essential oil compounds in *A*. *lancea* could vary depending on environmental factors such as biological and abiotic stress. In contrast, Takeda *et al*. [17] suggested that the contents of essential oil compounds are influenced largely by genetic factors based on a comparative study of the essential oil compounds between wild and cultivated *A*. *lancea*. However, extensive genetic analyses, particularly the estimation of heritability and Genotype-Environment (G × E) interaction analyses associated with the contents of the essential oil compounds in *A*. *lancea*, have not been performed.

In the present study, to evaluate effects of genetic factors on the contents of essential oil compounds in *A*. *lancea*, we cultivated twenty-five clonal lines of *A*. *lancea* in a micro-environment and estimated broad sense heritability for β-eudesmol, hinesol, atractylon, and atractylodin contents. In order to estimate the broad sense heritability, we employed the calculation model for vegetatively propagated crops such as orchardgrass, bromegrass, and tall fescue [18–20]. In addition, to examine G × E interaction and the effects of environmental factors, we cultivated six clones in different years (2016, 2017) and different locations (Hokkaido, Ibaraki), and determined contents of β-eudesmol, hinesol, atractylon, and atractylodin in the clones. Subsequently, we evaluated genetic variances, environmental variances, and G×E interaction variance based on the compound contents.

## Materials and methods

### Plant materials

In the present study, seeds of *A*. *lancea* obtained in a previous study were used [21]. The seeds were originally obtained from the People’s Republic of China and were from different genotypes. In other words, the seeds were harvested in natural mating. The plants were identified by the first author himself as *A*. *lancea* due to morphological characteristics. A clonal line of *A*. *lancea* was propagated from a rhizome of a single plant. Twenty-five clonal lines (line1–line25) with different genotypes were used in the present study.

### Cultivation of *A*. *lancea*

The 25 clonal lines were cultivated in an experimental field located in Ami-machi, Inashiki-gun, Ibaraki prefecture (35°.99’N, 140°.20’E), Japan, in 2017. Rhizomes of the 25 clonal lines were divided into about 50 g. The rhizomes were planted in November 25, 2016 and harvested on November 23, 2017. The cultivation was performed with 5–20 biological replicates for each clonal line. The replicates for each clonal line were as follows; line1–line17 (n = 20), line18–line24 (n = 10), line25 (n = 5).

To assess the effects of cultivation year on the contents of essential oil compounds in *A*. *lancea*, six clones (line1–line6) were also grown in the fields located in Ibaraki prefecture in 2016. The plants were cultivated from November 25, 2015 to November 23, 2016 in Ibaraki prefecture. The cultivation was performed with 20 biological replicates for each clonal line. The six clones were also cultivated in an experimental field located in Kyowa-town, Hokkaido prefecture (43°.01’N, 140°.53’E), Japan, in 2017, to evaluate the effects of cultivation location on the contents of essential oil compounds in *A*. *lancea*. Rhizomes of the plants were planted on October 21, 2016 and harvested on October 19, 2017. The plants were cultivated with 8–20 biological replicates for each clonal line. The replicates for each clonal lines were as follows; line1 (n = 20), line2 (n = 12), line3 (n = 8), line4 (n = 20), line5 (n = 18), line6 (n = 20).

### GC-MS analysis of essential oil compounds

The harvested rhizomes of *A*. *lancea* were dried in a convection oven at 50°C for 7 days and their dry weights were determined. The dried rhizomes were pulverized using a vibrating mill (Cosmic Mechanical Technology, TI-200). The contents of four essential oil compounds, namely, β-eudesmol, hinesol, atractylon, and atractylodin, were determined by gas chromatography-mass spectrometry (GC-MS) analysis as follows. Powdered samples of *A*. *lancea* rhizomes (0.5 g) were extracted with n-hexane (25 mL) for 15 min using a recipro shaker (Taitec, model SR-1) and centrifuged at 1660 × *g* for 10 min. The supernatant was separated, and the residue was re-extracted with n-hexane (20 mL) in a similar manner as above. The supernatant was combined, and phenanthrene (1.5 mg in 1 mL n-hexane) was added as an internal standard. A solution with a total volume of 50 mL was made by adding of n-hexane. An aliquot (1 μL) of the solution was injected into GC-MS. GC-MS analysis was carried out using an Agilent 7890 gas chromatograph equipped with a 5975 mass spectrum detector (MSD) (Agilent Technologies). Chromatography was performed with a DB-WAX GC column (polyethylene glycol, 30 m×250 μm i.d., 0.25 μm film; Agilent J&W Scientific). The column was used with the following temperature program: column held at 160°C for 2 min after injection, increased by 5°C/min to 200°C, then increased by 8°C/min to 240°C, and held for 5min. Injection temperature was set to 160°C. Helium was used as the carrier gas and the flow rate was 1.0 mL/min. The contents were calculated based on the dry weights of the powdered samples.

### Statistical analysis

Statistical analyses, including one-way ANOVA, two-way ANOVA, and correlation analysis (Pearson's correlation coefficient) were performed for β-eudesmol, hinesol, atractylon, and atractylodin contents using R (version 3.5.0). Broad sense heritability (h_B_) was estimated from variance components in ANOVA as follows: h_B_ = σ^2^_G_ / (σ^2^_G_ +σ^2^_E_), where σ^2^_G_ = genotypic variance, σ^2^_E_ = environmental variance [11, 20]. Effective number of replicates (r) for the estimation of σ^2^_G_ was calculated using the following formula: $r=(\sum_{i=1}^{a}r_{i}-\sum_{i=1}^{a}r_{i}^{2}/\sum_{i=1}^{a}r_{i})/(a-1)$, where a = the number of clonal lines [22].

## Results

### Estimation of broad sense heritability for the contents of essential oil compounds

**Fig 1:**
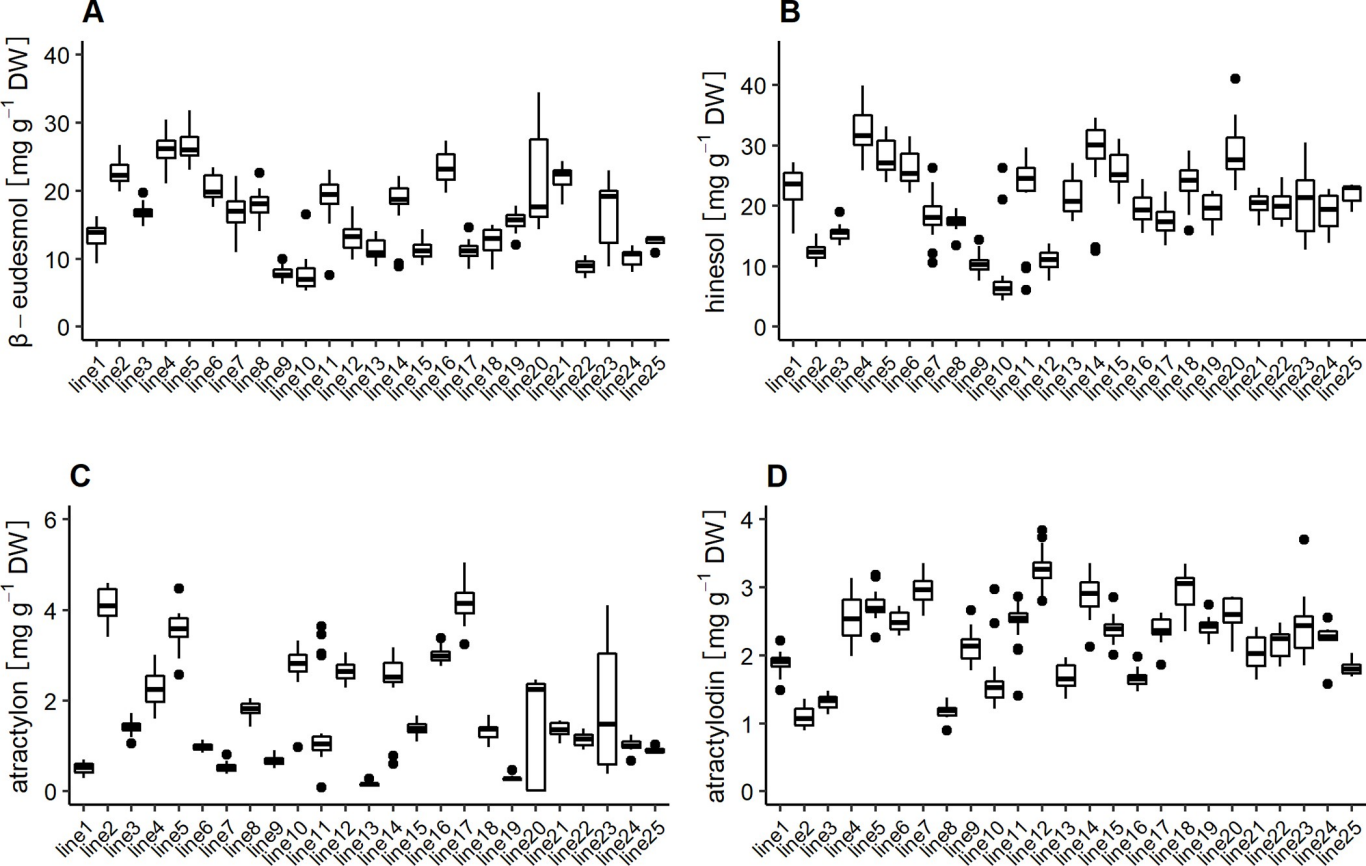
Range of variations for contents of the essential oil compounds in *A*. *lancea*. The boxes represent the values from the 25th to 75th percentile. The middle lines represent the median. The vertical lines extend from the minimum to the maximum values. Biological replicates within each clonal lines were as follows: line1–line17 (n = 20), line18–line24 (n = 10), line25 (n = 5). A; β-eudesmol contents, B; hinesol contents, C; atractylon contents, D; atractylodin contents.

To evaluate the heritability of the contents of essential oil compounds in *A*. *lancea* under a microenvironment, 25 clonal lines were grown in Ibaraki prefecture in 2017 and the contents of the compounds in individual plantlets were determined. Fig 1 illustrates the distribution of each compound in different clonal lines. In most clonal lines, the ranges of variation in the contents of β-eudesmol, hinesol, atractylon, and atractylodin were lower than varietal differences, although some lines exhibited relatively high variations among individual plantlets in the same line, for example, line20 and line23 (Fig 1). One-way ANOVA and the estimation of broad sense heritability of those compound contents were performed using the above results. Table 1 shows that the differences in varietal compound contents were significant (P > 0.01) and the heritability for the contents of β-eudesmol, hinesol, atractylon, and atractylodin were high at 0.84, 0.77, 0.86, and 0.87, respectively (Table 1).

**Table 1:** Broad-sense heritability for contents of the essential oil compounds in *A*. *lancea*.

Essential oil compounds	Factors	Df	Mean Sq	P-value	Effective replication	Genotypic variance	Environmental variance	Broad-sense heritability
**β-eudesmol**	Clonal lines	24	539.1	<2e-16**	16.5	32.2	6.3	0.84
	Residuals	390	6.3					
**hinesol**	Clonal lines	24	698.2	<2e-16**	16.5	41.5	12.7	0.77
	Residuals	390	12.7					
**atractylon**	Clonal lines	24	23.5	<2e-16**	16.5	1.4	0.2	0.87
	Residuals	390	0.2					
**atractylodin**	Clonal lines	24	6.1	<2e-16**	16.5	0.4	0.1	0.86
	Residuals	390	0.1					

Df; degree of freedom, Mean Sq; Mean square

**; Probability value for test of significance < 0.01.

**Fig 2:**
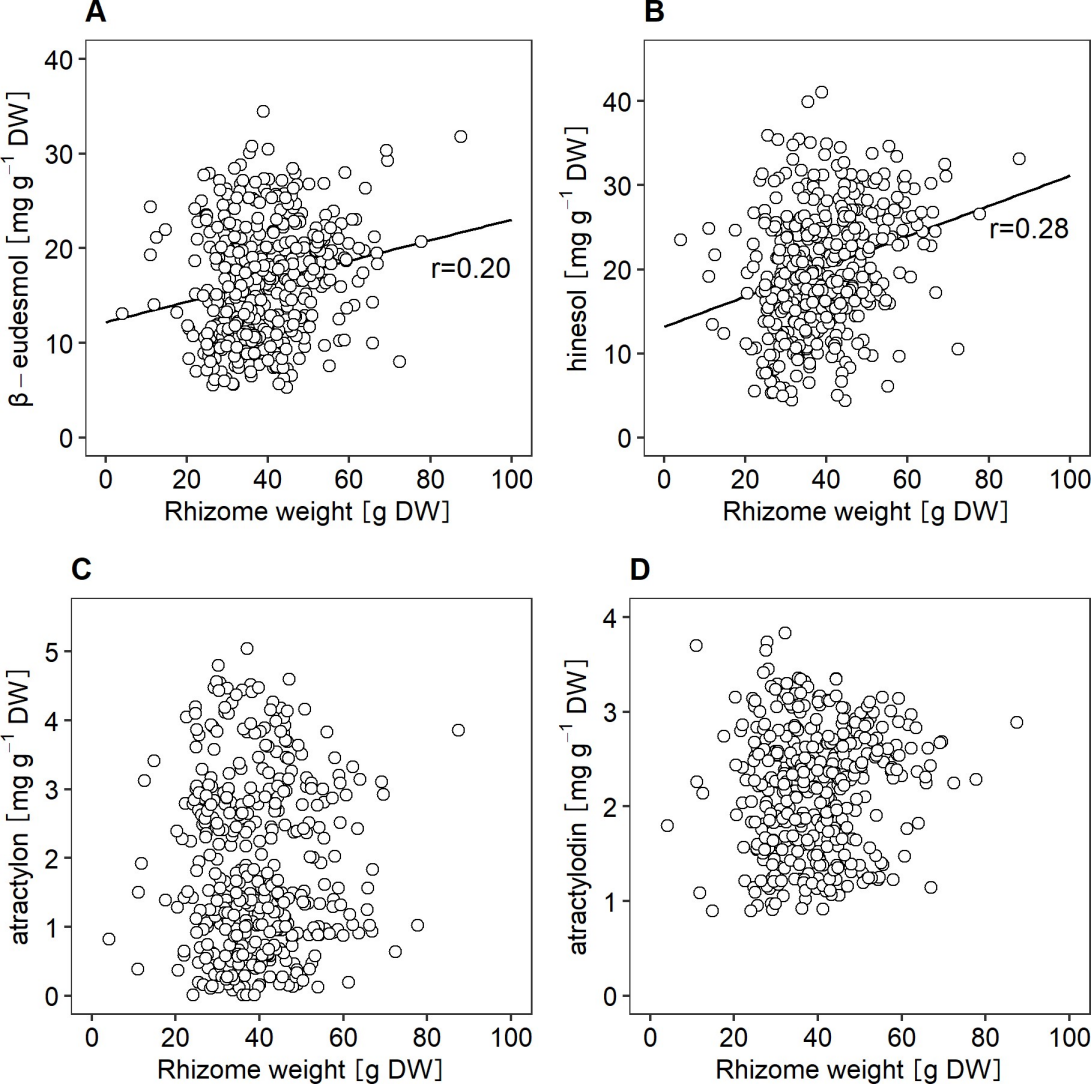
Relationships between contents of the essential oil compounds and rhizome weight in *A*. *lancea*. A; β-eudesmol contents, B; hinesol contents, C; atractylon contents, D; atractylodin contents.

Correlation analyses among various compound contents and rhizome yield were performed, since there is potential linkage among different traits. According to the results, there was no significant correlation between rhizome yields and contents of atractylon or atractylodin, and the correlation coefficient between rhizome yields and contents of β-eudesmol and hinesol were relatively low, 0.20 and 0.28, respectively (Fig 2). In contrast, the correlation between β-eudesmol and hinesol contents was relatively high, although the correlations among the other compounds were comparatively low (Table 2).

**Table 2:** Correlation matrix of β-eudesmol, hinesol, atractylon, and atractylodin contents.

	β-eudesmol	Hinesol	atractylon	atractylodin
**β-eudesmol**	-	0.59**	0.29**	0.09
**hinesol**	-	-	−0.13**	0.39**
**atractylon**	-	-	-	−0.04
**atractylodin**	-	-	-	-

**; Probability value for test of significance < 0.01.

### Environmental conditions of cultivation years and locations

Climatic datas for each cultivation years and locations were used the data issued by the Japan Meteorological Agency (JMA) [23]. Climatic conditions for the period of cultivation in Ibaraki prefecture were slightly different between 2016 and 2017. Mean temperatures in 2016 and 2017 were 14.9°C and 15.4°C, and accumulated rainfall in 2016 and 2017 were 1243 mm and 1196 mm, respectively. Environmental conditions of cultivation locations were more differed than that of cultivation years. Hokkaido is located in north of Japan, and mean temperature in Hokkaido is lower than mean temperature in Ibaraki prefecture. Mean temperatures for the period of cultivation in Hokkaido and Ibaraki prefecture were 15.4°C and 8.3°C, respectively. In addition, accumulated rainfall was differed between Hokkaido and Ibaraki prefecture. The accumulated rainfall for the period of cultivation in Hokkaido and Ibaraki prefecture was 1915 mm and 1487 mm, respectively. Further, there were difference in soil type and soil texture between 2 locations. The soil type and soil texture in Ibaraki prefecture were andosol and loam, and that in Hokkaido were alluvial soil and sandy loam.

### Effects of cultivation year on contents of the essential oil compounds

**Fig 3:**
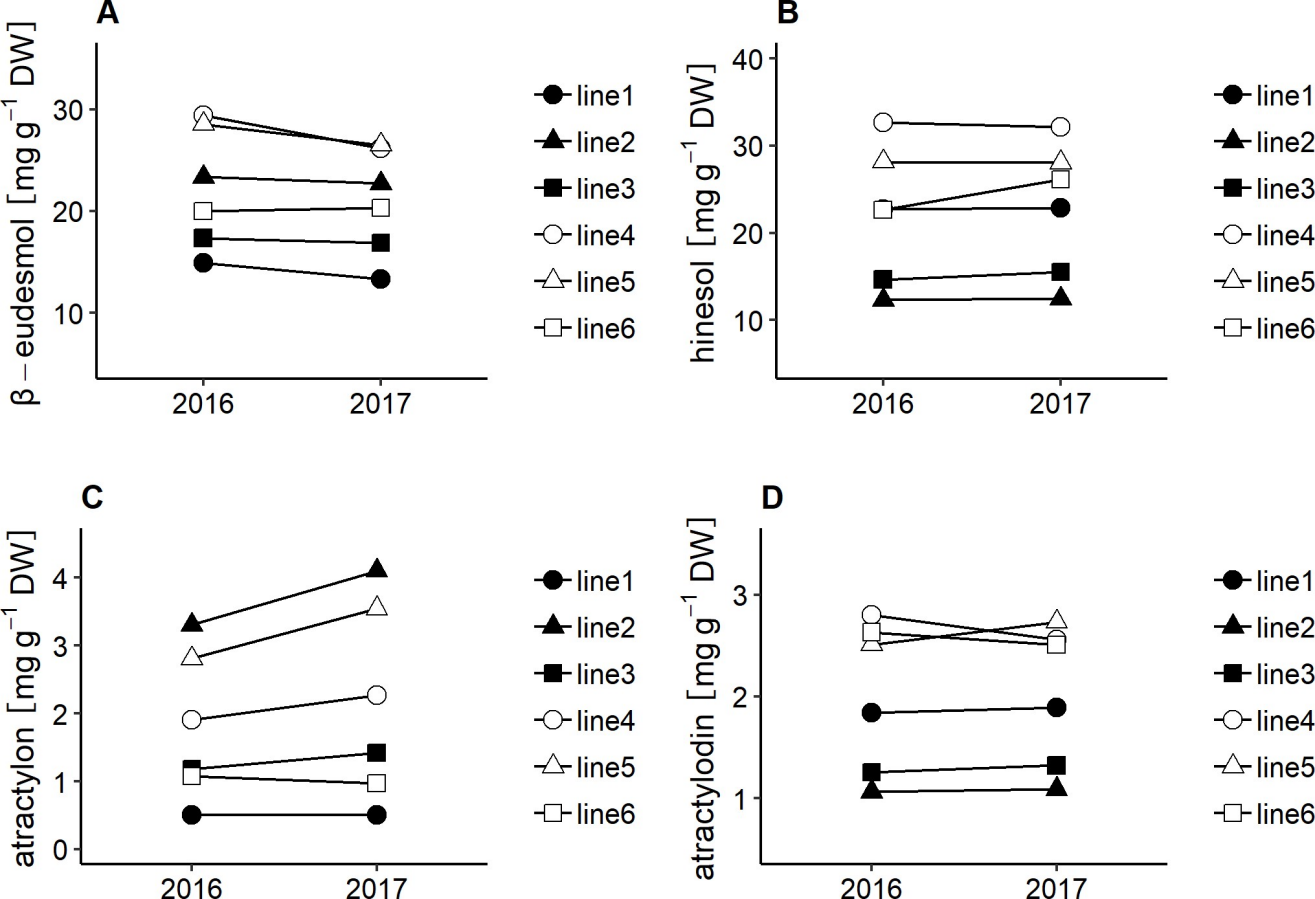
Interaction plots for interannual variability of the essential oil compound contents in *A*. *lancea*. Each point represents the mean of 20 measurements within clonal lines each in 2016 and 2017. A; β-eudesmol contents, B; hinesol contents, C; atractylon contents, D; atractylodin contents.

**Fig 4:**
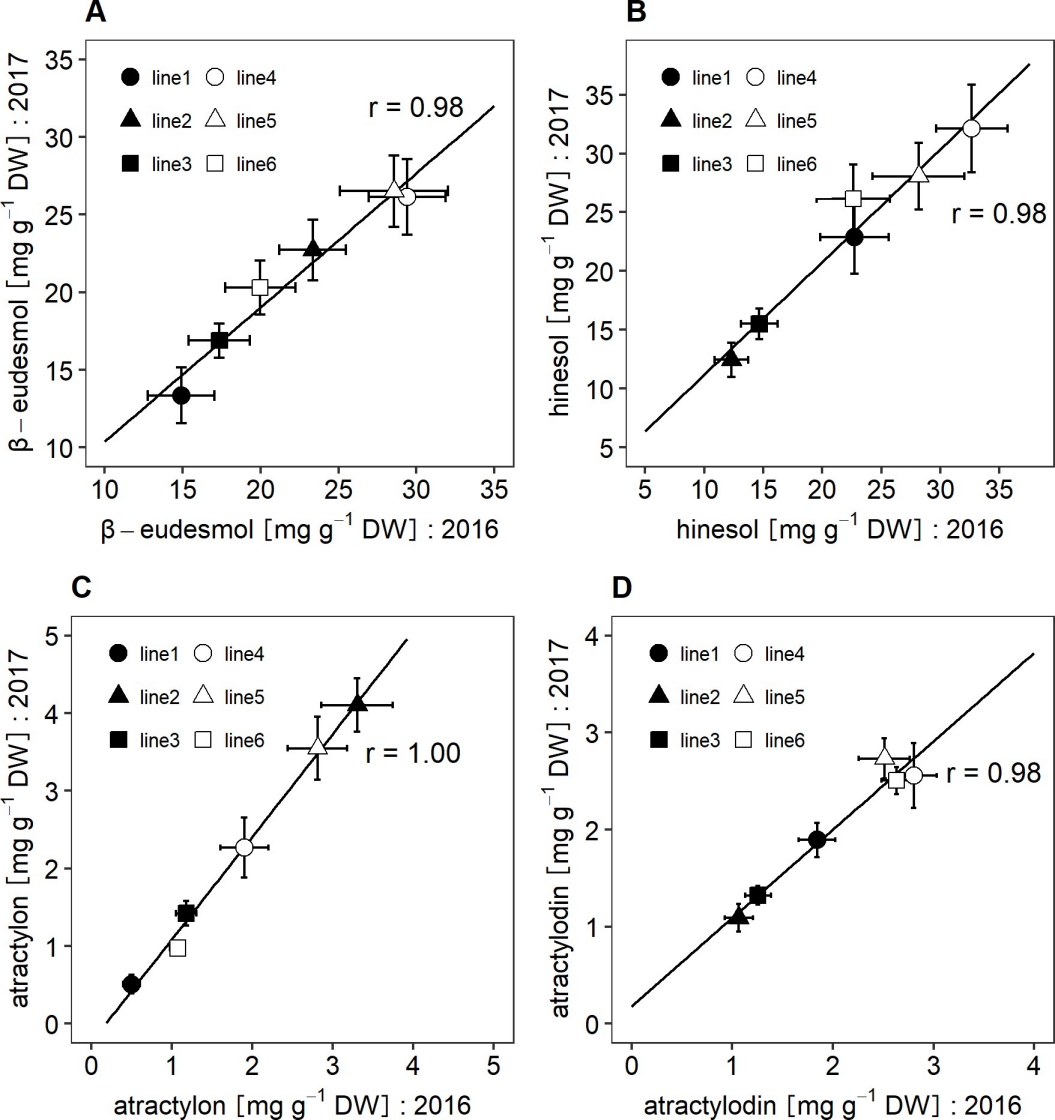
Relationships between contents of the essential oil compounds from six *A*. *lancea* clones grown in 2016 and 2017. Each point represents the mean of 20 measurements each in 2016 and 2017. A. β-eudesmol contents, B. hinesol contents, C. atractylon contents, D. atractylodin contents.

To examine the effect of cultivation year on contents of the essential oil compounds, six clones of *A*. *lancea* were grown in the same experimental field in 2016 and 2017, and contents of the compounds analyzed using two-way ANOVA. The effects of genotype (G), cultivation year (Y), and G × Y interaction were significant for contents of β-eudesmol, hinesol, and atractylon. However, mean squares of genotypes for each compound’s contents were higher than those of cultivation year and G × Y interaction (Table 3). In addition, interaction plots between genotype and cultivation year for β-eudesmol, hinesol, and atractylon showed that there were low qualitative interactions (Fig 3). Atractylodin contents were not significantly influenced by cultivation year, although there were significant differences for genotypes and G × Y interactions (Table 3). Fig 2D illustrates that qualitative interaction between genotype and cultivation year based on atractylodin contents was low, although in line5, the content levels of atractylodin in six clonal lines varied depending on cultivation year. In addition, the correlation coefficients between the two cultivation years for β-eudesmol, hinesol, atractylon, and atractylodin contents were 0.94, 0.94, 1.00, and 0.83, respectively (Fig 4).

**Table 3:** Two-way ANOVA results for β-eudesmol, hinesol, atractylon, and atractylodin contents in six *A*. *lancea* clones grown in 2016 and 2017.

Essential oil compounds	Factors	Df	MeanSq	P-value
**β-eudesmol**	Genotype (G)	5	12.26	2.0.E-16**
	Year (Y)	1	0.97	1.4.E-05**
	G × Y	5	0.16	6.1.E-03**
	Residuals	228	0.05	
**hinesol**	Genotype (G)	5	23.22	2.0E-16**
	Year (Y)	1	0.27	6.1E-02*
	G × Y	5	0.22	1.6E-02**
	Residuals	228	0.08	
**atractylon**	Genotype (G)	5	0.64	2.0.E-16**
	Year (Y)	1	0.07	2.0.E-16**
	G × Y	5	0.01	1.1.E-14**
	Residuals	228	0.001	
**atractylodin**	Genotype (G)	5	0.20	2.0E-16**
	Year (Y)	1	0.0.E + 00	0.9
	G × Y	5	0.003	2.6E-06**
	Residuals	228	0.0004	

Df; degree of freedom, Mean Sq; Mean square

*, **; Probability value for test of significance < 0.05 and < 0.01, respectively.

### Effects of cultivation location on the contents of essential oil compounds

**Fig 5:**
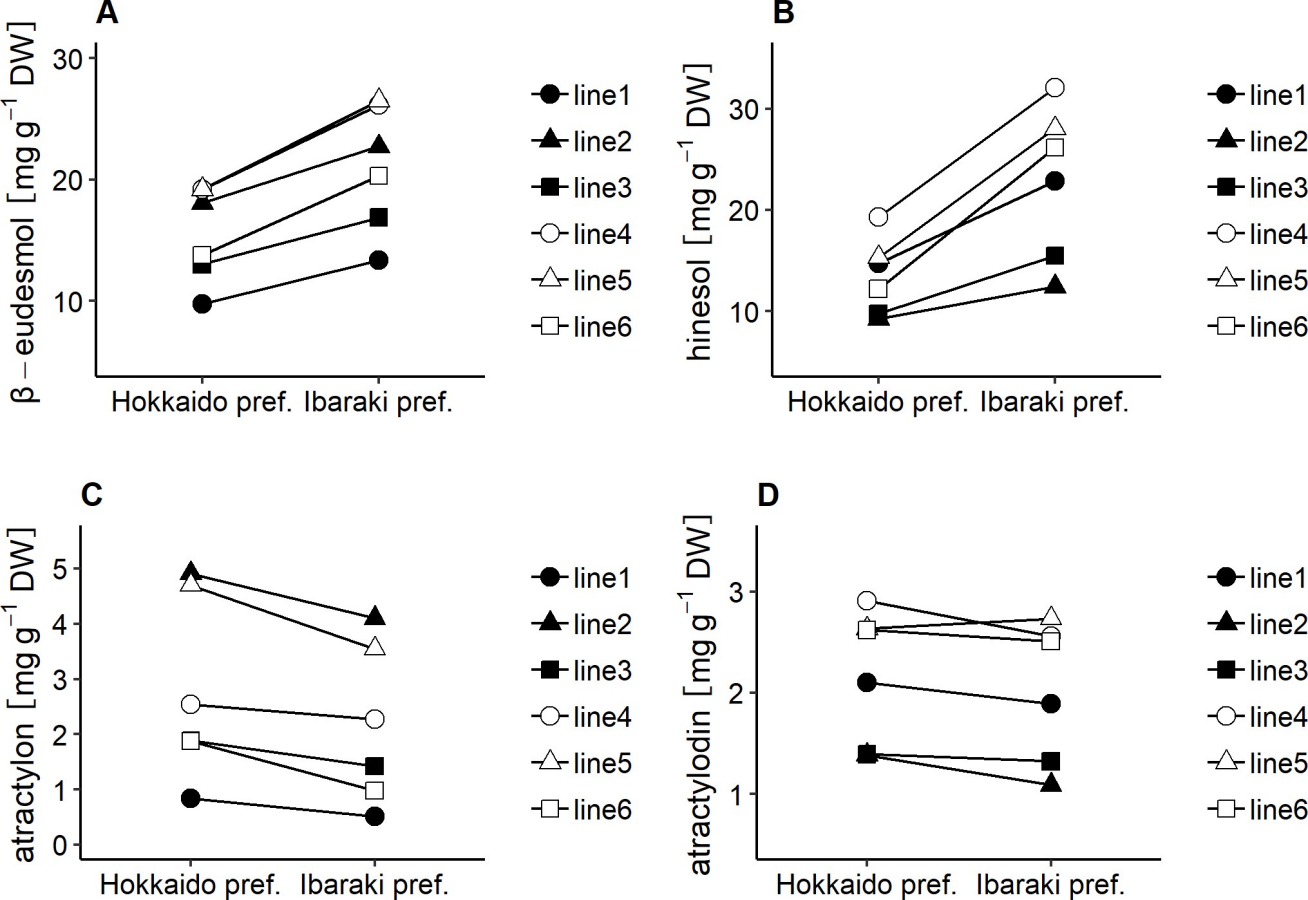
Interaction plots for contents of the essential oil compounds in six *A*. *lancea* clones grown in Hokkaido and Ibaraki prefectures. Data represent mean values of each compounds’ contents in *A*. *lancea* (n = 20 in line1, n = 12 in line2, n = 8 in line3, n = 20 in line4, n = 18 in line5, n = 20 in line6). A; β-eudesmol contents, B; hinesol contents, C; atractylon contents, D; atractylodin contents.

**Fig 6:**
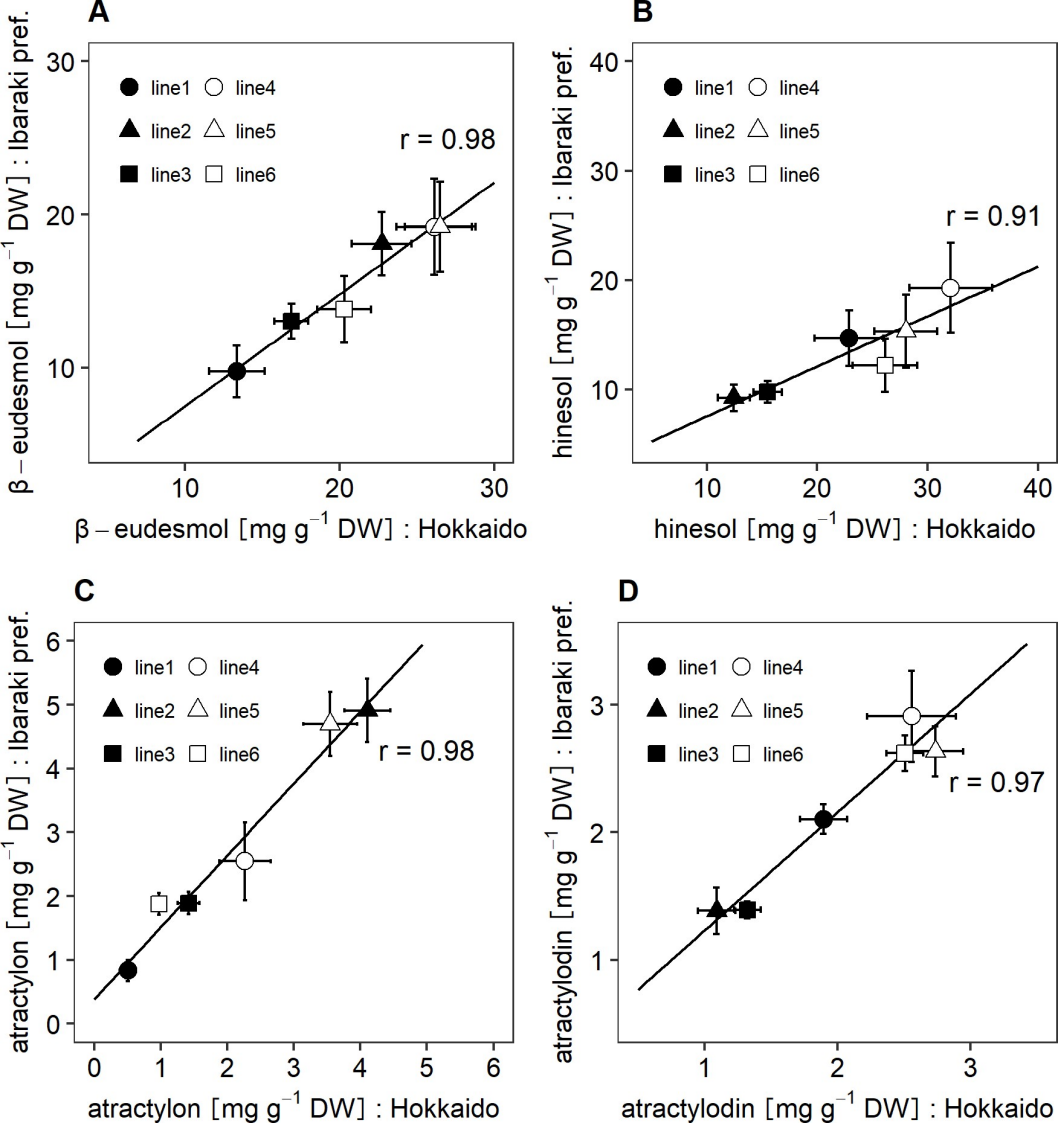
Relation for contents of the essential oil compounds from six *A*. *lancea* clones grown in Hokkaido and Ibaraki prefecture. Data represent means ± SD (n = 20 in line1, n = 12 in line2, n = 8 in line3, n = 20 in line4, n = 18 in line5, n = 20 in line6). A. β-eudesmol contents, B. hinesol contents, C. atractylon contents, D. atractylodin contents.

To determine the effects of cultivation location on contents of the essential oil compounds, six clonal lines of *A*. *lancea* were cultivated in different locations. Two-way ANOVA results for β-eudesmol and hinesol contents revealed that there were significant differences in genotypes (G), locations (L), and G × L interactions. In addition, mean squares of genotype were lower than those of location (Table 4). However, low qualitative interactions between genotype and location for contents of β-eudesmol and hinesol were observed (Fig 5). In addition, correlation coefficients for contents of β-eudesmol and hinesol between the two cultivation locations were 1.00 and 0.94, respectively (Fig 6).

**Table 4:** Two-way ANOVA for β-eudesmol, hinesol, atractylon, and atractylodin contents in six *A*. *lancea* clones grown in Hokkaido and Ibaraki prefecture.

Essential oil compounds	Factors	Df	MeanSq	P-value
**β-eudesmol**	Genotype (G)	5	7.80	2.0E-16**
	Location (L)	1	16.66	2.0E-16**
	G × L	5	0.24	2.2E-04**
	Residuals	206	0.05	
**hinesol**	Genotype (G)	5	9.61	2.0E-16**
	Location (L)	1	52.76	2.0E-16**
	G × L	5	1.58	2.3E-16**
	Residuals	206	0.08	
**atractylon**	Genotype (G)	5	0.83	2.0.E-16**
	Location (L)	1	0.23	2.0.E-16**
	G × L	5	0.01	1.9.E-08**
	Residuals	206	0.001	
**atractylodin**	Genotype (G)	5	0.16	2.0E-16**
	Location (L)	1	0.01	4.6E-08**
	G × L	5	0.002	3.9E-05**
	Residuals	206	0.0004	

Df; degree of freedom, Mean Sq; Mean square

**; Probability value for test of significance < 0.01.

Two-way ANOVA analyses of atractylon and atractylodin contents revealed that there were significant differences in genotypes, locations, and G × L interaction. However, the mean squares of genotypes were higher than those of locations and G × L interactions (Table 4). Fig 5C and 5D show that there were low qualitative interactions between genotype and location for contents of atractylon and atractylodin. In addition, correlation analyses of the contents of atractylon and atractylodin between the two locations revealed high correlations coefficients, 1.00 and 0.94, respectively (Fig 6).

## Discussion

In the present study, broad sense heritability for β-eudesmol, hinesol, atractylon, and atractylodin contents in *A*. *lancea* was high (Table 1), suggesting that the contents of the essential oil compounds in *A*. *lancea* are highly influenced by genetic factors. In addition, correlations between each compound contents and rhizome yield were low (Fig 2), indicating that *A*. *lancea* strains, which have high contents of essential oil compounds could be selectively bred irrespective of rhizome yields. Therefore, cultivars of *A*. *lancea* with not only high contents of essential oil compounds but also high yields could be developed through selective breeding.

Correlation coefficients among different compound contents indicated low correlations, except in the case of the correlation between β-eudesmol and hinesol contents (Table 2). The biosynthetic pathways of β-eudesmol and hinesol are considered potentially closely associate since the compounds have similar chemical structures with the same molecular mass [24]. In addition, Takeda *et al*. [21] observed that the content ratio of β-eudesmol to hinesol keep constant in *A*. *lancea* clones. The above results could be the reason why the correlation coefficient between β-eudesmol and hinesol contents was high. When selectively breeding for the contents of the compounds, that the contents of β-eudesmol and hinesol could be linked. Conversely, the contents of the other compounds could be selected independently in the course of cultivar development.

We used the six clonal lines to investigate the influence of cultivation year on the contents of the essential oil compounds. Based on the results of the two-way ANOVA analyses, mean squares of genotype for contents of the essential oil compounds were higher than the interannual variability (Table 3). Additionally, there were low G × Y qualitative interactions (Fig 3), while the correlation coefficients between cultivation years and compound contents were relatively high (Fig 4). The results imply that, regardless of cultivation year, the contents of essential oil compounds in *A*. *lancea* clonal lines were stable, and genetic factors had greater influence than cultivation year did.

We cultivated six clones in two locations and evaluated the effects of cultivation location on the contents of essential oil compounds. The effect of cultivation location on the contents of essential oil compounds in *A*. *lancea* was larger than that of cultivation year, especially, the mean squares of cultivation location for β-eudesmol and hinesol contents were higher than those of genotype (Table 4). In general, secondary metabolites such as sesquiterpenes are induced by biological and abiotic stress via phytohormone signaling [25]. In *A*. *lancea*, the contents of essential oil compounds are induced by phytohormones such as jasmonic acid and ABA, via symbiosis with endophytes [14, 15]. In addition, increased soil acidity triggers an increase in β-eudesmol contents [16]. Therefore, the influence of cultivation location on the accumulation of β-eudesmol and hinesol in the present study could be induced by plant hormones due to biological or abiotic stress. It has been reported that mean temperature in their natural habitats are 11.1–16.0°C, and accumulated rainfall are 850–1560 mm [13]. In the present study, there were differences in climatic and soil conditions between the 2 cultivation locations. Mean temperatures in Ibaraki were within the range of mean temperature for the natural habitats, whereas the average temperature of Hokkaido was lower than that of the natural habitats. It is possible that the contents of essential oil compounds in *A*. *lancea* could be influenced by these climatic differences. Furthermore, it has been reported that soil type and soil cray content are ecological factors having the high contribution rate against atractylodin contents in *A*. *lancea* [26]. In the present study, soil type and soil texture were different between the 2 cultivation locations, and the contents of the essential oil compounds in *A*. *laneca* might be varied by these soil conditions. More detailed analyses on the influence of environmental factors are required, particularly on what are the major factors influencing the contents of the essential oil compounds.

Conversely, low qualitative interactions between genotype and location in the contents of β-eudesmol, hinesol, atractylon and atractylodin were observed (Fig 5), and correlation coefficients between 2 locations for these componds’ contents were high (Fig 6). The results suggest that genetic potential of *A*. *lancea* regarding the contents of β-eudesmol, hinesol, atractylon and atractylodin are stable regardless of environmental conditions, although the contents of the compounds are influenced by environmental factors as absolute values. It has been reported that artemisinin contents in *Artemisia annua* clones grown under different cultivation conditions were highly correlated [27]. The contents of β-eudesmol, hinesol, atractylon, and atractylodin in *A*. *lancea* exhibited similar results in the present study. The results indicate that the sesquiterpenoids and polyacetylene content levels among genotypes are not influenced by environmental conditions, although their absolute values varied depending on environments. Therefore, selective breeding could be effective in controlling the contents of essential oil compounds in *A*. *lancea*. In addition, cultivars exhibiting wide adaptation in the contents of essential oil compounds could be developed. Recent studies have proposed a putative sesquiterpenoid biosynthetic pathway based on transcriptome analyses of different *A*. *lancea* tissues, and several candidate genes related to sesquiterpenoid biosynthesis have been reported [28, 29]. As for sesquiterpenoid biosynthesis, β-eudesmol synthase mainly expressed in rhizomes has been identified in other plants such as *Zingiber zerumbet* [30]. Furthermore, high levels of SNPs (simple nucleotide polymorphisms) have been reported from leaf, stem, and root cDNA libraries of *A*. *lancea* [31]. The results suggest the potential of generating genetic markers for the contents of essential oil compounds in *A*. *lancea* using genetic association analysis.

In conclusion, the present study demonstrates that the contents of β-eudesmol, hinesol, atractylon, and atractylodin in *A*. *lancea* are influenced mainly by genetic factors, and selective breeding could be an effective strategy for developing *A*. *lancea* populations that yield high contents of essential oil compounds. In addition, β-eudesmol, hinesol, atractylon, and atractylodin contents could be selected regardless of rhizome yields in the course of *A*. *lancea* cultivar development. Consequently, *A*. *lancea* cultivars with high rhizome yields and high contents of each the essential oil compounds could be developed. However, since the contents of the essential oil compounds are influenced by environmental condition, further investigations on the effects of environment factors on the compound contents in *A*. *lancea* are required.

## Supporting information

S1 Table The data for Figs 1 and 2 and Tables 1 and 2. The contents of essential oil compounds in *A*. *lancea* grown in Ibaraki prefecture on 2017.
(PDF)

S2 Table The data for Figs 3 and 4 and Table 3. The contents of essential oil compounds in *A*. *lancea* grown in each cultivation year.
(PDF)

S3 Table The data for Figs 5 and 6 and Table 4. The contents of essential oil compounds in *A*. *lancea* grown in each cultivation location.
(PDF)
