# Supplementary material for: Genetic and environmental factors influencing the contents of essential oil compounds in Atractylodes lancea
Source: PLoS One. 2019 May 28;14(5):e0217522. doi: 10.1371/journal.pone.0217522 (PMC6538177; doi:10.1371/journal.pone.0217522)
Supplement: S1 Table — The contents of essential oil compounds in A. lancea grown in Ibaraki prefecture on 2017. (PDF) [file pone.0217522.s001.pdf]

S1 Table. The data for Fig.1-2 and Table 1- 2. The contents of essential oil compounds in A. lancea grown in Ibaraki prefecture on 2017.

clonal line No.	The contents of essential oil compounds / cultivation year : 2017, cultivation location : Ibaraki				
	β−eudesmol (mg/gDW)	hinesol (mg/gDW)	atractylon (mg/gDW)	atractylodin (mg/gDW)	Rhizome weight (g DW)
line1	16.30	27.18	0.67	2.21	39.16
line1	12.01	21.38	0.41	2.05	27.62
line1	14.65	24.58	0.30	1.93	36.48
line1	14.50	24.56	0.65	1.94	45.42
line1	13.97	23.10	0.40	1.91	39.05
line1	10.56	18.09	0.71	2.22	25.78
line1	12.43	21.65	0.31	1.65	29.23
line1	12.29	21.76	0.59	1.91	42.23
line1	12.42	21.14	0.53	1.74	42.48
line1	9.33	15.41	0.63	1.95	28.63
line1	12.27	20.14	0.61	1.49	29.11
line1	14.15	25.57	0.60	2.00	27.52
line1	14.28	25.44	0.42	1.85	30.73
line1	13.86	25.20	0.56	1.80	36.01
line1	15.23	26.76	0.60	2.04	22.01
line1	14.73	25.48	0.52	1.85	35.85
line1	13.96	24.06	0.41	1.91	32.91
line1	11.53	19.58	0.38	1.92	20.47
line1	16.06	25.78	0.42	1.86	39.79
line1	12.39	20.93	0.47	1.66	48.18
line2	20.89	11.19	3.84	1.03	36.97
line2	23.75	12.31	4.18	1.07	45.04
line2	22.60	12.51	4.55	1.37	30.57
line2	21.57	12.35	4.48	1.25	29.15
line2	26.74	14.44	4.60	1.16	47.01
line2	21.52	12.63	4.52	1.25	37.91
line2	24.58	14.97	3.91	0.98	28.80
line2	26.30	15.39	4.57	1.27	29.63
line2	19.87	10.90	3.94	1.13	28.44
line2	20.95	10.66	4.05	1.22	22.49
line2	23.47	12.52	3.58	0.92	29.15
line2	25.30	13.72	3.88	0.92	36.16
line2	21.10	11.31	4.13	1.06	31.72
line2	22.54	11.79	3.84	0.97	29.76
line2	22.02	12.38	3.41	0.90	14.72
line2	20.18	9.86	4.45	1.23	35.81
line2	21.81	11.40	3.89	0.92	41.20
line2	21.74	11.80	3.79	0.96	25.59
line2	24.09	13.03	4.27	1.07	43.12
line2	23.48	13.81	4.20	1.15	24.62
line3	15.77	14.66	1.20	1.13	39.67
line3	15.70	13.95	1.73	1.46	40.22
line3	16.12	14.95	1.57	1.43	40.39
line3	14.83	13.83	1.39	1.35	32.04
line3	18.35	15.95	1.62	1.39	55.52
line3	16.78	15.42	1.39	1.48	38.87
line3	16.68	15.93	1.34	1.23	54.28
line3	16.24	13.44	1.67	1.41	39.59
line3	16.59	14.50	1.50	1.31	47.86
line3	16.48	15.55	1.28	1.43	33.36
line3	16.93	16.13	1.28	1.24	39.98
line3	18.63	15.86	1.52	1.30	50.55
line3	17.74	16.99	1.05	1.20	30.47
line3	16.21	14.05	1.49	1.29	37.70

line3	16.75	15.87	1.36	1.23	44.30
line3	16.62	15.39	1.45	1.36	44.86
line3	17.14	16.16	1.46	1.38	49.83
line3	16.79	15.61	1.43	1.38	41.17
line3	19.68	19.06	1.35	1.24	51.67
line3	17.62	16.83	1.34	1.20	50.74
line4	23.57	27.03	3.01	3.06	24.75
line4	25.76	31.43	1.99	2.58	29.86
line4	27.22	34.53	1.90	2.20	43.41
line4	25.35	30.16	2.63	2.82	35.07
line4	28.81	35.48	2.19	2.82	33.15
line4	26.81	35.50	1.61	1.99	33.17
line4	26.07	29.68	2.82	3.11	33.06
line4	25.08	30.23	2.18	2.29	36.66
line4	21.07	26.04	2.33	2.46	33.37
line4	26.28	31.24	2.52	2.84	39.01
line4	28.46	34.83	2.79	3.14	31.74
line4	27.93	35.93	1.95	2.61	25.49
line4	27.22	31.83	2.40	2.72	33.70
line4	30.46	34.92	2.25	2.63	40.02
line4	22.37	25.89	2.65	2.28	37.17
line4	27.18	35.41	2.04	2.17	28.05
line4	30.09	39.95	1.83	2.49	35.36
line4	25.06	30.62	2.25	2.39	23.58
line4	24.24	33.11	1.66	2.15	31.68
line4	23.70	28.28	2.38	2.38	48.18
line5	23.11	27.07	3.27	2.69	47.54
line5	26.78	28.27	3.51	2.74	49.97
line5	28.04	30.73	3.21	2.55	58.95
line5	29.29	31.09	2.93	2.69	69.44
line5	26.18	30.90	2.58	2.27	28.07
line5	30.37	32.51	3.11	2.67	69.11
line5	23.48	25.99	3.62	2.68	24.81
line5	27.33	28.15	3.85	3.16	40.09
line5	25.72	26.38	3.47	2.72	39.72
line5	25.78	27.16	3.54	2.55	43.96
line5	24.26	24.31	3.47	2.63	46.24
line5	24.71	27.07	3.46	2.64	46.84
line5	27.93	30.45	3.69	2.80	46.61
line5	23.95	23.92	3.84	2.57	56.06
line5	31.84	33.19	3.86	2.89	87.42
line5	25.33	25.14	4.47	3.19	32.21
line5	25.71	24.66	3.62	2.67	41.96
line5	25.56	25.75	3.81	2.88	45.78
line5	28.44	31.29	3.93	2.94	46.02
line5	26.35	27.10	3.72	2.73	46.13
line6	17.63	22.42	1.15	2.39	32.04
line6	18.12	22.23	1.04	2.49	39.67
line6	22.23	28.51	1.01	2.72	27.56
line6	19.42	26.79	0.92	2.37	57.45
line6	18.79	24.37	0.96	2.63	45.10
line6	23.49	31.37	0.88	2.63	54.23
line6	19.81	23.52	0.89	2.56	51.81
line6	19.55	24.55	0.94	2.43	66.78
line6	19.82	25.79	0.96	2.36	44.15
line6	18.05	24.30	1.03	2.48	39.25
line6	22.80	29.62	0.85	2.43	47.80
line6	19.03	23.18	0.97	2.65	58.69
line6	19.74	23.62	0.97	2.47	55.62
line6	19.16	24.92	1.08	2.59	38.33
line6	20.51	26.19	0.88	2.32	59.85

line6	19.74	24.56	0.92	2.37	63.57
line6	20.73	26.58	1.03	2.29	77.73
line6	22.61	29.02	1.05	2.73	46.74
line6	22.46	30.10	1.01	2.73	54.08
line6	22.24	31.49	0.96	2.51	43.71
line7	15.02	15.54	0.49	3.05	33.86
line7	16.83	19.01	0.50	3.08	40.40
line7	17.52	18.05	0.48	2.94	52.45
line7	15.38	17.72	0.63	2.68	34.23
line7	16.61	17.73	0.44	2.75	32.40
line7	16.26	16.64	0.81	3.36	35.25
line7	19.87	23.92	0.47	2.58	30.37
line7	10.94	10.57	0.67	2.81	35.12
line7	18.89	19.57	0.45	2.83	38.31
line7	20.61	23.16	0.39	2.93	48.82
line7	17.14	19.21	0.47	3.17	40.43
line7	15.98	16.95	0.58	3.00	53.16
line7	18.29	22.88	0.42	3.11	40.05
line7	14.39	15.22	0.48	2.84	25.33
line7	17.64	19.27	0.51	3.20	34.57
line7	15.30	17.01	0.59	3.13	42.23
line7	12.97	12.16	0.60	2.67	42.34
line7	18.86	20.86	0.53	3.08	33.36
line7	22.18	26.24	0.39	2.88	51.86
line7	17.32	18.03	0.51	3.04	46.33
line8	15.59	16.24	1.82	1.10	25.00
line8	19.38	16.29	1.94	1.26	54.57
line8	22.61	19.61	2.03	1.23	57.94
line8	14.08	13.50	1.93	1.09	11.80
line8	19.79	18.32	2.01	1.25	52.69
line8	18.68	18.07	1.79	1.23	34.35
line8	15.91	16.18	1.90	1.23	36.73
line8	20.35	19.13	2.02	1.25	52.06
line8	18.06	17.57	1.83	1.20	36.61
line8	16.79	16.51	1.74	1.11	37.01
line8	15.05	17.42	1.43	0.90	23.97
line8	18.38	17.26	1.84	1.15	66.95
line8	17.76	18.76	1.79	1.11	41.91
line8	17.71	18.04	1.84	1.19	38.58
line8	20.04	17.82	2.06	1.38	40.49
line8	18.05	17.28	1.55	1.17	41.10
line8	19.01	18.57	1.53	1.16	27.82
line8	18.70	17.82	1.60	1.12	42.82
line8	16.79	16.95	1.70	1.18	30.54
line8	16.82	17.19	1.77	1.21	29.37
line9	8.96	13.41	0.60	2.20	36.85
line9	7.72	10.06	0.71	2.22	37.79
line9	9.62	11.59	0.91	2.67	49.02
line9	7.44	9.76	0.66	2.07	42.94
line9	7.28	8.95	0.68	2.23	44.68
line9	8.52	11.29	0.58	2.10	42.36
line9	7.41	9.63	0.74	2.06	41.24
line9	6.50	8.43	0.74	1.87	35.89
line9	7.87	10.80	0.67	2.33	35.03
line9	6.29	8.12	0.55	1.82	27.16
line9	6.56	8.32	0.63	1.87	45.71
line9	8.37	11.20	0.73	2.28	47.69
line9	8.05	10.52	0.64	2.25	72.43
line9	8.83	11.01	0.51	2.17	33.28
line9	7.59	9.94	0.58	1.97	31.85
line9	6.29	7.68	0.68	1.78	43.86

line9	7.47	10.96	0.60	2.15	41.94
line9	9.98	14.36	0.74	2.46	43.41
line9	7.54	10.60	0.62	1.90	41.99
line9	7.40	9.65	0.74	2.12	32.76
line10	7.01	5.52	2.80	1.57	22.27
line10	8.63	7.36	3.00	1.64	32.53
line10	8.94	7.72	2.79	1.84	24.14
line10	9.94	8.49	3.18	1.58	28.33
line10	8.35	6.66	2.45	1.44	26.99
line10	8.64	7.75	2.70	1.55	25.34
line10	6.28	5.91	2.94	1.45	29.54
line10	5.63	4.45	2.96	1.54	31.36
line10	7.00	6.00	3.04	1.65	28.50
line10	7.66	6.69	3.29	1.61	26.28
line10	16.56	26.27	3.33	2.97	62.22
line10	7.00	6.54	3.30	1.39	35.62
line10	5.55	5.34	2.49	1.22	26.38
line10	5.30	4.39	2.69	1.38	44.48
line10	5.67	5.06	2.46	1.24	42.68
line10	7.40	6.70	2.76	1.33	43.65
line10	6.12	5.38	2.87	1.51	26.83
line10	5.67	5.47	2.41	1.22	31.20
line10	6.01	4.97	2.84	1.38	29.15
line10	16.52	21.09	0.98	2.47	34.97
line11	19.79	26.14	0.08	2.54	33.55
line11	19.72	23.20	1.27	2.81	30.53
line11	18.51	24.71	0.75	2.56	27.51
line11	19.74	24.52	0.88	2.67	47.70
line11	16.38	9.79	3.04	2.10	51.19
line11	18.79	22.51	1.15	2.55	45.95
line11	21.65	27.13	0.91	2.61	56.64
line11	20.01	25.28	1.03	2.62	62.23
line11	20.89	25.79	0.92	2.52	53.66
line11	23.05	29.62	1.18	2.86	61.49
line11	15.94	9.67	3.46	2.30	57.85
line11	18.16	23.42	0.79	2.46	38.99
line11	19.09	24.47	1.19	2.53	36.07
line11	7.63	6.13	3.00	1.41	54.98
line11	17.87	23.63	1.05	2.50	35.38
line11	21.26	26.84	1.03	2.62	66.21
line11	15.19	10.04	3.64	2.08	49.22
line11	18.89	22.24	1.17	2.64	45.87
line11	20.91	27.02	0.99	2.70	52.95
line11	21.40	27.79	0.89	2.54	53.95
line12	14.94	12.49	2.43	3.32	36.87
line12	11.37	8.86	2.78	3.00	35.76
line12	14.93	12.52	3.07	3.74	27.79
line12	11.84	9.87	2.52	2.82	27.01
line12	10.61	8.99	2.85	3.15	24.31
line12	11.69	9.82	2.64	3.00	26.10
line12	13.57	11.18	2.41	3.29	30.31
line12	9.90	7.67	3.05	3.12	24.99
line12	11.28	9.27	2.66	3.15	32.83
line12	14.01	12.23	2.77	3.46	28.10
line12	14.54	11.64	2.66	3.34	35.83
line12	17.72	13.79	2.87	3.84	32.14
line12	10.80	10.51	2.28	2.80	21.53
line12	13.37	11.14	2.46	3.26	37.60
line12	12.71	10.68	3.01	3.30	32.48
line12	11.86	11.23	2.39	3.16	20.22
line12	13.29	12.03	2.59	3.27	28.84

line12	14.94	13.01	2.58	3.42	26.95
line12	13.28	11.09	2.51	3.24	29.83
line12	14.33	13.51	2.69	3.65	27.48
line13	11.05	21.41	0.16	1.65	34.52
line13	10.65	19.98	0.28	1.97	30.93
line13	10.77	20.49	0.17	1.84	32.35
line13	9.34	19.09	0.20	1.56	25.33
line13	10.49	19.09	0.23	1.96	48.83
line13	14.03	26.35	0.20	1.77	61.16
line13	10.67	19.48	0.14	1.64	28.74
line13	8.89	17.52	0.13	1.60	36.33
line13	13.42	24.65	0.17	1.76	26.51
line13	9.24	17.70	0.12	1.53	35.15
line13	12.93	24.16	0.18	1.65	48.96
line13	12.69	24.92	0.14	1.40	47.89
line13	9.75	18.25	0.13	1.90	30.12
line13	9.78	18.29	0.13	1.91	53.82
line13	11.98	20.93	0.17	1.53	39.98
line13	12.83	24.20	0.16	1.78	46.80
line13	13.86	27.07	0.18	1.36	44.76
line13	10.96	21.57	0.11	1.57	28.20
line13	10.62	19.04	0.15	1.92	28.71
line13	11.95	23.92	0.14	1.49	39.75
line14	22.16	34.63	2.82	3.15	55.34
line14	18.64	30.45	2.78	2.99	50.22
line14	18.39	29.91	2.48	2.77	46.04
line14	18.10	28.37	2.41	2.68	43.06
line14	16.35	24.71	2.45	2.59	54.16
line14	20.29	32.40	2.29	3.12	42.67
line14	21.06	33.43	2.88	3.01	57.28
line14	21.44	33.82	3.17	2.73	51.14
line14	18.22	27.55	2.68	2.71	43.60
line14	18.68	29.79	2.52	2.90	59.23
line14	18.84	27.93	3.09	3.15	59.17
line14	20.11	31.18	2.40	2.89	48.87
line14	18.08	29.81	2.37	3.07	36.76
line14	20.57	32.76	2.89	3.35	44.24
line14	9.34	13.13	0.79	2.52	38.98
line14	17.41	26.85	2.43	2.83	63.31
line14	20.18	30.31	2.52	3.06	51.69
line14	8.92	12.55	0.61	2.13	34.90
line14	20.13	33.65	2.79	2.92	39.55
line14	20.66	30.80	3.09	3.11	52.30
line15	12.51	30.06	1.23	2.01	44.36
line15	11.70	26.87	1.37	2.36	50.24
line15	11.56	24.02	1.50	2.19	45.23
line15	10.29	24.55	1.65	2.61	35.12
line15	10.49	26.38	1.67	2.86	34.42
line15	10.25	22.90	1.40	2.41	57.55
line15	9.10	20.31	1.31	2.17	34.52
line15	10.35	23.90	1.32	2.16	37.50
line15	11.47	23.42	1.16	2.30	28.77
line15	11.08	25.40	1.45	2.41	35.37
line15	10.43	24.08	1.35	2.43	36.73
line15	12.53	28.57	1.30	2.40	57.44
line15	10.79	24.92	1.42	2.45	46.91
line15	11.16	26.71	1.48	2.33	44.32
line15	10.00	22.83	1.57	2.25	65.76
line15	10.35	24.65	1.57	2.39	58.82
line15	14.34	30.23	1.25	2.31	65.68
line15	11.90	29.04	1.39	2.48	37.96

line15	13.70	31.06	1.33	2.54	59.31
line15	12.59	28.34	1.10	2.47	36.71
line16	20.15	15.50	2.96	1.60	25.56
line16	27.35	24.49	2.89	1.69	38.86
line16	25.55	21.34	3.01	1.66	42.25
line16	24.71	19.24	3.16	1.58	44.78
line16	26.87	23.71	2.77	1.48	53.78
line16	25.35	20.42	3.38	1.65	35.19
line16	22.42	18.81	2.88	1.68	32.12
line16	25.38	22.28	3.05	1.66	44.23
line16	23.06	19.10	2.92	1.48	60.60
line16	22.89	17.85	2.98	1.49	46.26
line16	24.86	20.77	3.03	1.60	37.86
line16	23.22	19.80	2.84	1.65	37.48
line16	21.70	18.49	2.90	1.84	41.32
line16	22.59	19.31	2.80	1.75	43.69
line16	19.71	17.43	2.91	1.54	27.30
line16	21.25	17.56	3.02	1.57	52.25
line16	25.33	21.66	3.30	1.79	40.78
line16	26.37	23.00	3.15	1.83	63.92
line16	20.75	16.65	2.97	1.66	45.71
line16	20.06	16.29	3.16	1.98	46.92
line17	12.30	20.70	4.11	2.44	32.50
line17	14.60	22.44	4.42	2.46	38.14
line17	8.51	13.47	4.27	2.52	33.41
line17	11.28	17.53	4.05	2.34	45.72
line17	12.92	20.21	4.16	2.30	50.74
line17	10.71	17.18	5.05	2.63	37.02
line17	11.05	17.45	3.87	2.53	25.02
line17	10.47	16.06	4.19	2.53	31.64
line17	11.46	17.53	3.82	2.21	38.86
line17	10.37	15.63	4.38	2.39	34.41
line17	12.80	21.00	3.65	2.31	41.43
line17	10.02	15.46	3.99	2.36	43.82
line17	11.17	16.25	4.48	2.19	39.66
line17	10.16	15.37	3.24	1.86	42.21
line17	11.08	16.26	4.80	2.54	30.07
line17	10.19	15.61	4.43	2.55	30.31
line17	11.76	17.33	3.84	2.29	45.00
line17	10.66	17.56	3.96	2.33	37.89
line17	11.34	18.58	4.15	2.37	42.60
line17	12.92	20.17	4.14	2.32	46.54
line18	11.56	23.79	1.19	3.06	30.15
line18	11.20	22.11	0.97	2.46	34.32
line18	9.83	18.47	1.13	2.74	28.55
line18	8.38	15.95	1.22	2.36	43.41
line18	13.41	24.36	1.41	3.20	35.83
line18	13.25	24.64	1.39	2.75	17.44
line18	12.71	24.05	1.38	3.06	35.24
line18	14.54	26.30	1.68	3.17	44.33
line18	14.99	29.14	1.50	3.34	44.22
line18	14.54	26.29	1.36	3.05	45.77
line19	16.62	21.85	0.28	2.33	48.60
line19	15.45	19.57	0.24	2.48	33.81
line19	14.70	17.27	0.34	2.42	48.85
line19	16.07	21.59	0.30	2.57	33.36
line19	16.01	19.44	0.28	2.29	45.38
line19	17.82	22.49	0.27	2.75	42.42
line19	15.16	19.74	0.24	2.35	32.00
line19	12.09	15.14	0.28	2.17	32.26
line19	17.12	22.14	0.22	2.44	50.30

line19	13.73	15.81	0.47	2.48	35.01
line20	30.80	35.10	0.02	2.44	35.91
line20	17.83	26.68	2.37	2.85	51.16
line20	14.32	22.60	2.43	2.47	50.51
line20	17.40	28.54	2.46	2.84	34.37
line20	27.77	31.37	0.02	2.05	24.07
line20	27.00	31.22	0.02	2.52	36.90
line20	34.52	41.05	0.02	2.63	38.73
line20	15.16	25.08	2.36	2.87	27.19
line20	15.94	26.34	2.21	2.57	30.23
line20	16.70	25.95	2.30	2.84	55.27
line21	24.23	23.00	1.43	2.42	21.96
line21	24.41	19.16	1.51	2.26	11.05
line21	18.32	17.29	1.06	1.67	27.41
line21	23.08	21.51	1.28	1.94	41.97
line21	20.81	21.98	1.16	1.83	29.46
line21	21.20	19.71	1.28	1.88	31.61
line21	22.79	21.56	1.51	2.28	22.73
line21	22.67	20.99	1.57	2.26	30.49
line21	17.99	16.79	1.26	1.64	29.30
line21	22.28	20.09	1.50	2.10	34.44
line22	8.34	17.17	1.29	2.44	20.43
line22	9.48	21.33	1.26	2.48	28.12
line22	7.34	16.55	0.97	2.23	26.51
line22	9.69	19.52	1.39	2.27	29.82
line22	9.38	21.81	1.01	1.96	44.47
line22	8.00	18.98	1.09	2.06	32.53
line22	10.53	24.76	1.03	1.88	36.99
line22	7.18	17.49	0.92	1.84	24.88
line22	8.63	20.39	1.21	2.32	31.74
line22	9.60	21.65	1.21	2.27	32.34
line23	19.34	24.86	0.39	3.70	10.93
line23	22.98	25.56	4.10	2.48	24.71
line23	19.59	21.06	3.24	1.92	32.75
line23	8.84	12.73	0.58	2.10	34.78
line23	20.13	22.30	2.78	1.85	28.45
line23	18.69	20.32	0.65	2.87	21.97
line23	10.20	14.32	0.60	2.39	36.01
line23	18.98	30.52	2.31	2.61	27.04
line23	21.16	21.75	3.12	2.14	12.45
line23	9.63	13.63	0.59	2.47	40.58
line24	8.93	16.14	0.97	2.21	36.54
line24	8.06	13.86	1.25	2.56	25.82
line24	10.82	19.73	0.92	2.26	36.54
line24	10.66	19.52	1.01	2.36	36.53
line24	10.97	19.33	1.18	2.34	33.81
line24	8.14	14.31	1.04	2.38	39.91
line24	9.86	18.21	1.12	2.28	24.85
line24	10.89	22.63	0.68	1.58	42.09
line24	11.83	22.31	0.97	2.23	31.87
line24	11.98	22.79	1.01	2.23	46.18
line25	12.95	23.08	1.03	2.04	40.59
line25	13.05	20.90	0.87	1.69	33.99
line25	13.14	23.56	0.82	1.80	3.97
line25	10.90	19.03	0.88	1.86	34.61
line25	12.31	22.97	0.93	1.73	42.48
