# Supplementary material for: Genetic and environmental factors influencing the contents of essential oil compounds in Atractylodes lancea
Source: PLoS One. 2019 May 28;14(5):e0217522. doi: 10.1371/journal.pone.0217522 (PMC6538177; doi:10.1371/journal.pone.0217522)
Supplement: S3 Table — The contents of essential oil compounds in A. lancea grown in each cultivation location. (PDF) [file pone.0217522.s003.pdf]

S3 Table. The data for Fig.5-6 and Table 4. The contents of essential oil compounds in A. lancea grown in each cultivation location.

clonal line No.	compound contents / cultivation location							
	eudesmol (mg/g DW)		hinesol (mg/g DW)		atractylodin (mg/g DW)		atractylon (mg/g DW)	
	Ibaraki pref.	Hokkaido	Ibaraki pref.	Hokkaido	Ibaraki pref.	Hokkaido	Ibaraki pref.	Hokkaido
line1	16.30	12.55	27.18	17.14	2.21	2.30	0.67	0.85
line1	12.01	11.10	21.38	16.38	2.05	2.16	0.41	0.62
line1	14.65	8.36	24.58	14.37	1.93	1.81	0.30	0.65
line1	14.50	11.02	24.56	15.59	1.94	2.16	0.65	1.00
line1	13.97	6.97	23.10	10.78	1.91	2.06	0.40	0.88
line1	10.56	11.42	18.09	16.49	2.22	2.15	0.71	0.94
line1	12.43	10.59	21.65	16.41	1.65	2.15	0.31	0.83
line1	12.29	10.67	21.76	15.24	1.91	2.10	0.59	0.90
line1	12.42	8.93	21.14	13.61	1.74	2.02	0.53	0.90
line1	9.33	10.68	15.41	15.12	1.95	2.16	0.63	0.92
line1	12.27	10.07	20.14	13.92	1.49	2.28	0.61	1.05
line1	14.15	10.20	25.57	16.80	2.00	2.06	0.60	0.62
line1	14.28	12.41	25.44	20.07	1.85	2.23	0.42	0.62
line1	13.86	8.29	25.20	11.99	1.80	1.95	0.56	0.79
line1	15.23	7.78	26.76	11.43	2.04	1.98	0.60	1.01
line1	14.73	7.15	25.48	10.80	1.85	2.06	0.52	0.94
line1	13.96	7.88	24.06	10.89	1.91	2.19	0.41	1.09
line1	11.53	10.10	19.58	16.30	1.92	2.09	0.38	0.51
line1	16.06	10.98	25.78	16.98	1.86	2.14	0.42	0.73
line1	12.39	8.09	20.93	13.51	1.66	1.99	0.47	0.83
line2	20.89	18.26	11.19	8.72	1.03	1.73	3.84	5.72
line2	23.75	19.83	12.31	10.44	1.07	1.63	4.18	5.54
line2	22.60	19.55	12.51	10.36	1.37	1.26	4.55	4.37
line2	21.57	23.02	12.35	11.87	1.25	1.38	4.48	5.30
line2	26.74	17.85	14.44	8.88	1.16	1.50	4.60	5.19
line2	21.52	16.49	12.63	8.29	1.25	1.47	4.52	5.09
line2	24.58	15.26	14.97	7.66	0.98	1.10	3.91	4.33
line2	26.30	17.91	15.39	9.50	1.27	1.33	4.57	4.84
line2	19.87	17.91	10.90	9.50	1.13	1.33	3.94	4.84
line2	20.95	18.53	10.66	9.50	1.22	1.36	4.05	4.67
line2	23.47	16.57	12.52	7.88	0.92	1.39	3.58	4.96
line2	25.30	15.88	13.72	8.36	0.92	1.15	3.88	4.08
line2	21.10		11.31		1.06		4.13	
line2	22.54		11.79		0.97		3.84	
line2	22.02		12.38		0.90		3.41	
line2	20.18		9.86		1.23		4.45	
line2	21.81		11.40		0.92		3.89	
line2	21.74		11.80		0.96		3.79	
line2	24.09		13.03		1.07		4.27	
line2	23.48		13.81		1.15		4.20	
line3	15.77	12.52	14.66	9.24	1.13	1.49	1.20	2.05
line3	15.70	13.09	13.95	10.35	1.46	1.29	1.73	1.75
line3	16.12	12.71	14.95	9.35	1.43	1.43	1.57	1.85
line3	14.83	14.12	13.83	10.32	1.35	1.36	1.39	1.90
line3	18.35	13.77	15.95	10.23	1.39	1.41	1.62	2.02
line3	16.78	11.12	15.42	9.16	1.48	1.32	1.39	1.69
line3	16.68	14.63	15.93	11.30	1.23	1.40	1.34	1.70
line3	16.24	12.21	13.44	8.11	1.41	1.44	1.67	2.17
line3	16.59		14.50		1.31		1.50	
line3	16.48		15.55		1.43		1.28	
line3	16.93		16.13		1.24		1.28	
line3	18.63		15.86		1.30		1.52	
line3	17.74		16.99		1.20		1.05	
line3	16.21		14.05		1.29		1.49	
line3	16.75		15.87		1.23		1.36	
line3	16.62		15.39		1.36		1.45	
line3	17.14		16.16		1.38		1.46	
line3	16.79		15.61		1.38		1.43	
line3	19.68		19.06		1.24		1.35	
line3	17.62		16.83		1.20		1.34	
line4	23.57	18.77	27.03	21.08	3.06	2.64	3.01	2.32
line4	25.76	18.80	31.43	18.47	2.58	3.04	1.99	2.27
line4	27.22	23.49	34.53	26.66	2.20	2.36	1.90	1.22
line4	25.35	14.95	30.16	14.42	2.82	2.68	2.63	2.80

line4	28.81	19.95	35.48	20.49	2.82	2.45	2.19	1.69
line4	26.81	19.08	35.50	20.48	1.99	2.58	1.61	2.02
line4	26.07	19.41	29.68	16.35	3.11	3.64	2.82	3.73
line4	25.08	18.29	30.23	16.84	2.29	3.10	2.18	3.22
line4	21.07	22.74	26.04	23.88	2.46	3.16	2.33	2.60
line4	26.28	18.99	31.24	20.19	2.84	2.89	2.52	1.95
line4	28.46	14.83	34.83	14.68	3.14	2.83	2.79	3.09
line4	27.93	13.52	35.93	13.91	2.61	2.42	1.95	2.28
line4	27.22	15.69	31.83	13.02	2.72	3.00	2.40	3.04
line4	30.46	25.07	34.92	26.82	2.63	2.76	2.25	1.89
line4	22.37	20.64	25.89	20.81	2.28	3.09	2.65	2.70
line4	27.18	19.04	35.41	19.39	2.17	2.71	2.04	2.86
line4	30.09	16.70	39.95	14.85	2.49	3.19	1.83	3.34
line4	25.06	23.42	30.62	24.58	2.39	3.57	2.25	2.60
line4	24.24	22.60	33.11	21.23	2.15	3.28	1.66	2.87
line4	23.70	17.83	28.28	17.73	2.38	2.78	2.38	2.43
line5	23.11	21.20	27.07	18.36	2.69	2.62	3.27	4.44
line5	26.78	14.65	28.27	8.83	2.74	2.72	3.51	5.63
line5	28.04	19.79	30.73	14.82	2.55	2.97	3.21	5.26
line5	29.29	17.55	31.09	13.24	2.69	2.94	2.93	5.48
line5	26.18	15.09	30.90	10.80	2.27	2.63	2.58	4.77
line5	30.37	22.66	32.51	19.80	2.67	2.70	3.11	4.59
line5	23.48	22.72	25.99	19.06	2.68	2.33	3.62	4.07
line5	27.33	23.59	28.15	18.24	3.16	2.88	3.85	5.16
line5	25.72	17.47	26.38	14.73	2.72	2.58	3.47	4.71
line5	25.78	14.82	27.16	10.65	2.55	2.78	3.54	5.12
line5	24.26	18.61	24.31	15.70	2.63	2.54	3.47	4.72
line5	24.71	18.74	27.07	14.76	2.64	2.67	3.46	4.88
line5	27.93	18.28	30.45	15.55	2.80	2.59	3.69	4.45
line5	23.95	23.55	23.92	21.31	2.57	2.55	3.84	3.97
line5	31.84	19.28	33.19	16.16	2.89	2.70	3.86	4.55
line5	25.33	16.06	25.14	12.36	3.19	2.36	4.47	4.36
line5	25.71	21.67	24.66	14.28	2.67	2.23	3.62	3.80
line5	25.56	19.97	25.75	16.94	2.88	2.63	3.81	4.63
line5	28.44		31.29		2.94		3.93	
line5	26.35		27.10		2.73		3.72	
line6	17.63	15.06	22.42	13.47	2.39	2.74	1.15	1.97
line6	18.12	13.26	22.23	11.40	2.49	2.53	1.04	1.77
line6	22.23	12.69	28.51	10.87	2.72	2.48	1.01	1.89
line6	19.42	15.41	26.79	13.77	2.37	2.60	0.92	1.86
line6	18.79	14.40	24.37	12.43	2.63	2.66	0.96	1.87
line6	23.49	13.70	31.37	12.51	2.63	2.61	0.88	1.77
line6	19.81	12.24	23.52	9.43	2.56	2.68	0.89	2.03
line6	19.55	15.34	24.55	14.65	2.43	2.51	0.94	1.59
line6	19.82	10.76	25.79	9.32	2.36	2.55	0.96	1.96
line6	18.05	12.45	24.30	11.76	2.48	2.39	1.03	1.62
line6	22.80	18.58	29.62	18.04	2.43	2.64	0.85	1.59
line6	19.03	11.15	23.18	9.72	2.65	2.54	0.97	1.95
line6	19.74	15.36	23.62	13.65	2.47	2.62	0.97	1.84
line6	19.16	14.56	24.92	13.92	2.59	2.59	1.08	1.78
line6	20.51	8.94	26.19	7.17	2.32	2.40	0.88	1.91
line6	19.74	12.94	24.56	11.34	2.37	2.68	0.92	2.07
line6	20.73	13.65	26.58	10.73	2.29	2.80	1.03	2.12
line6	22.61	16.08	29.02	12.93	2.73	2.99	1.05	2.23
line6	22.46	13.35	30.10	11.57	2.73	2.57	1.01	1.78
line6	22.24	16.17	31.49	15.37	2.51	2.79	0.96	1.94
